# An Epigenetic Aging Clock for Cattle Using Portable Sequencing Technology

**DOI:** 10.3389/fgene.2021.760450

**Published:** 2021-11-18

**Authors:** Ben J. Hayes, Loan T. Nguyen, Mehrnush Forutan, Bailey N. Engle, Harrison J. Lamb, James P. Copley, Imtiaz A. S. Randhawa, Elizabeth M. Ross

**Affiliations:** ^1^ Centre for Animal Science, Queensland Alliance for Agriculture and Food Innovation, University of Queensland, St Lucia, QLD, Australia; ^2^ School of Veterinary Science, University of Queensland, Gatton, QLD, Australia

**Keywords:** cattle, epigenetic clock, long-read sequencing, age prediction, DNA methylation

## Abstract

Extensively grazed cattle are often mustered only once a year. Therefore, birthdates are typically unknown or inaccurate. Birthdates would be useful for deriving important traits (growth rate; calving interval), breed registrations, and making management decisions. Epigenetic clocks use methylation of DNA to predict an individual’s age. An epigenetic clock for cattle could provide a solution to the challenges of industry birthdate recording. Here we derived the first epigenetic clock for tropically adapted cattle using portable sequencing devices from tail hair, a tissue which is widely used in industry for genotyping. Cattle (*n* = 66) with ages ranging from 0.35 to 15.7 years were sequenced using Oxford Nanopore Technologies MinION and methylation was called at CpG sites across the genome. Sites were then filtered and used to calculate a covariance relationship matrix based on methylation state. Best linear unbiased prediction was used with 10-fold cross validation to predict age. A second methylation relationship matrix was also calculated that contained sites associated with genes used in the dog and human epigenetic clocks. The correlation between predicted age and actual age was 0.71 for all sites and 0.60 for dog and human gene epigenetic clock sites. The mean absolute deviation was 1.4 years for animals aged less than 3 years of age, and 1.5 years for animals aged 3–10 years. This is the first reported epigenetic clock using industry relevant samples in cattle.

## Introduction

Despite the success of genomic prediction in cattle, which has driven high rates of genetic gain in some livestock industries, there are still some challenges with implementation of genomic selection, especially in extensive beef cattle operations. Accurate recording of age of animals can be a significant challenge, particularly in extensive environments where animals may only be mustered once a year. This has adverse implications for both herd management and the estimation of genomic breeding values for economically important traits reliant on accurate age records, such as growth rate and age at puberty, reducing industry adoption of genetic and genomic evaluations.

Recent studies in humans have shown that aging is related to the alteration of DNA methylation in genome specific locations, and these epigenetic modifications can be used to estimate the individual’s age (Horvath, 2013; Weidner et al., 2014; Xu et al., 2015). Epigenetic clocks using methylation markers have reported accurate age prediction in humans and mice (Horvath, 2013; Horvath and Raj, 2018). Additionally, a DNA methylation clock has been constructed for dogs (*n* = 46) and wolves (*n* = 62) using blood samples (Thompson et al., 2017). The mean absolute deviation (MAD) of predicted age using linear regression models are species and sample size dependent, and likely related to the species lifespan (Yi et al., 2014; Zbieć-Piekarska et al., 2015; Stubbs et al., 2017; Li et al., 2018). While human methylomic studies mostly use methylation arrays for identifying CpGs sites, other epigenetic clocks in animals (dogs, wolves and chicken) have used bisulfite-treated sequencing approaches.

In cattle, epigenetic clocks have been constructed to estimate the biological age of oocytes using the HorvathMammalian40K array which contains 37,000 mammalian CpGs sites (Kordowitzki et al., 2021). Still, there is a lack of industry relevant methods available that could be efficiently and cost-effectively used in commercial applications that do not compromise animal health or wellbeing. Critically, epigenetic clocks based on samples that can be easily obtained in industry have not been developed.

Here we aimed to derive the first epigenetic clock for indicine cattle and their crosses using portable long-read sequencing. We used 66 samples from Brahman, Tropical Composite, and Droughtmaster breeds of cattle with a wide range of ages, spanning a few weeks to 15.7 years. To the best of our knowledge, this is the first cattle specific epigenetic clock using Oxford Nanopore Technology (ONT; Oxford, United Kingdom) sequencing platforms from an industry focused sample.

## Methods

Animal ethics approval was obtained from the University of Queensland ethics board (Animal Ethics Approval Number QAAFI/269/17).

### Sample Selection

**FIGURE 1:**
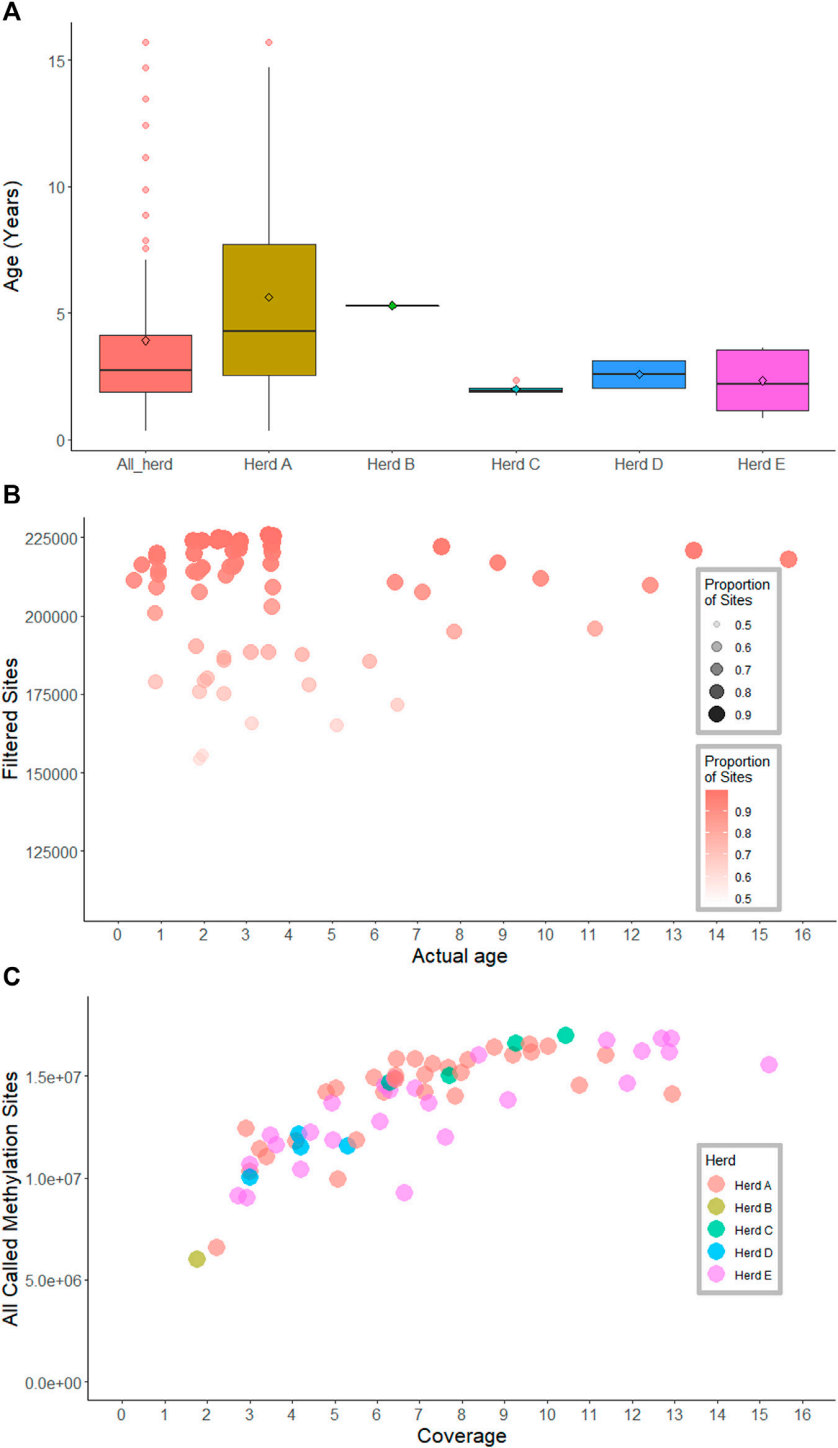
Age, methylation sites and sequencing coverage information across samples. **(A)** boxplots show age across herds and within-herd. **(B)** Scatterplot of age, methylation sites and proportion of sites presented in the methylation matrices. **(C)** Sequence coverage and the total number of methylated sites called for 66 samples.

Animals (all female) from industry were used in this study to ensure the accuracies were reflective of what could be expected in the field and were not biased by controlled laboratory conditions. Samples were selected so the methylation clock could be used to predict across a wide range of ages (Table 1; Figure 1A). Focus was placed on young cattle, where age often differs by only a few weeks, increasing our power to predict small differences in age within these cohorts. Animals from several breeds commonly raised in northern Australia, including Brahman, Droughtmaster and Tropical Composite, were included to ensure that the derived clock was not breed-specific (Table 1). Tail hair was selected as the source of the genetic material as it is easily collected by cattle producers. More information about animal age and the season of sample collection was provided in Supplementary Table S1.

**TABLE 1:** Characteristics of samples used for methylation analysis.

Herd	Breed	Birth date (Years)	Age (years)	Independent samples	Repeat samples	Calves	Total samples	Sequencing depth (mean ± SD)
Herd A	Droughtmaster	2001 to 2019	0.35–15.7	27	4	0	31	18.5 ± 7.2
Herd B	Brahman	2011 to 2013	5.30	1	0	0	1	4.75
Herd C	Tropical Composite	2016 and 2017	1.75–2.35	4	0	0	4	22.8 ± 4.9
Herd D	Droughtmaster	2015 and 2016	2.00–3.13	4	0	0	4	11.3 ± 2.5
Herd E	Brahman	2016 and 2020	0.83–3.64	5	14	7	26	20.2 ± 9.9
-	-	-	Total	41	18	7	66	-

### DNA Extraction and Sequencing

Genomic DNA was extracted using the Gentra Puregene Tissue Kit (Qiagen, Hilden, Germany) according to the manufacturer’s instructions, with modifications. Briefly, 20–30 hair samples were lysed in 300 µl of Cell lysis solution (Gentra^®^ Puregene^®^ Tissue Kit) and 1.5 µl of Proteinase K solution (20 mg/ml) for 5 h at 55°C. RNA was then digested by addition of 1.5 µl of RNase A Solution, following 1 h incubation at 37°C. Samples were placed on ice for 5 min after adding 100 µl Protein Precipitation Solution (Gentra^®^ Puregene^®^ Tissue Kit) and spun at 14,000 × g for 3 min. 300 µl of Isopropanol was used to precipitate DNA. Samples were centrifuged at 14,000 × g for 3 min. DNA pellets were washed in 300 ml of 70% ethanol, air-dried for 5 min, and resuspended in 55 µl of DNA Hydration Solution (Gentra^®^ Puregene^®^ Tissue Kit). DNA concentrations were measured using the Qubit dsDNA Broad Range assay kit (Thermo Fisher Scientific). The purity of the extracted DNA was determined with the NanoDropND 1000 (v.3.5.2, Thermo Fisher Scientific), assessing the 260/280 nm and 260/230 nm ratios. The size of extracted DNA was examined using pulsed-field gel electrophoresis (Sage science, United States) with a 0.75% Seakem Gold agarose gel (Lonza, United States) in 0.5X Tris/Borate/EDTA (TBE) running buffer, run for 16 h at 75 V. The gel was stained after the electrophoresis with SYBR Safe dye (10000x) and visualized using Quantity One analysis software (Bio-rad).

Extracted DNA samples were prepared using a ligation kit (SQK-LSK 109; ONT, Oxford, United Kingdom) as described in Lamb et al. (2020). Libraries were loaded onto the minION flow-cell (R.9.4.1; ONT, Oxford, United Kingdom) and run for up to 96 h. The flow-cell was washed at least three times using the nuclease-flush kit (ONT, Oxford, United Kingdom) and then reloaded with the same library solution.

### Methylation Calling

Base calls were made from the raw current disruption data using GUPPY (version 4.2.2) on the University of Queensland high performance computing infrastructure. Long reads were mapped to *Bos taurus* reference genome (ARS-UCD1.2 assembly) using minimap2 (version 2.17) with -x map-ont option (Li, 2018; Rosen et al., 2020). F5c (Gamaarachchi et al., 2020) was then used to implement GPU based methylation calling. Methylation frequency was subsequently calculated using f5c software.

### Testing of Conserved Age-Related Genes

The homologes of the genes that were associated with aging in dogs and humans (Wang et al., 2020) were identified in the *Bos taurus* genome annotation file. Methylation within each of those genes was then calculated for each sample as the (C_m_/C) * 100; where C_m_ is the number of methylated CpG sites within the gene on all of the overlapping reads, and C is the number of all CpG sites (both methylated and unmethylated) within the gene. Genes that did not have sequence coverage in more than 10 samples were excluded from the analysis.

In order to investigate the association of each of these genes with cattle age, we used the linear mixed model (fitted with the R package lme4, Bates et al., 2015):

age∼μ+methylation+herd

Where age is the age of the animal in years, µ is the overall mean, methylation is the percentage of methylated CpG sites in the gene being tested, and herd is a random effect accounting for the animals’ herd of origin. *P*-values for the model were calculated using lmerTest (Kuznetsova et al., 2017) and the coefficient of determination (marginal pseudo-R-squared value) was calculated using MuMIn (Bartoń, 2020; statistics based on; Nakagawa et al., 2017). *p*-values were only considered as significant if the direction of the effect was the same as those reported in Wang et al. (2020).

### Age Prediction

To precinct the age of animals similarities between the genome wide methylation patterns were used. First, a methylation score was derived for each genome segment of each animal. For each 100 base pair window across the whole genome, each window for each animal was called as methylated (1) or not methylated (0) if the average frequency of methylation for the sites in the window was greater than 0.5 or less than 0.5, respectively. This resulted in a matrix that contained 0, 1 or NA (where no sequencing coverage was present). The resulting matrix was then filtered to remove sites which were not called in at least 80% of animals. Sites that were not variable in the dataset, as indicated by a standard deviation less than 0.5, were also removed from the matrix. There was no correlation between the proportion of sites presented in the matrix per sample and age (Figure 1B, Supplementary Figure S1).

Using the methylation call matrix, methylation relationship matrices (MRM) were constructed among the animals. Two MRM were considered: 1) Using all sites that passed the filtering criteria for missing values and variation (called MRM-1); 2) Using only sites within genes reported as age predictive in both humans and dogs (Wang et al., 2020; called MRM-2). Importantly, all genes associated with age in humans and dogs from Wang et al. (2020), not only the ones associated with age in our own data, were used to generate MRM-2 to avoid artificially inflating the prediction accuracy based on preselection of genes within the same dataset. There were 226,600 sites and 65,137 sites used to calculate MRM-1 and MRM-2, respectively. Each of the relationship matrices was calculated by: **MRM = XX**’/(number of sites), where **X** is MRM-1 or MRM-2 (a matrix of animal by number of sites).

Methylation best linear unbiased prediction (MeBLUP) was used to predict age from the nanopore methylation profiles. Season was tested as a fixed effect in the model to separate the environmental stress effects on methylation profiles from the chronological effect (and did not improve accuracy of prediction). However, the impact of season was not significant. Therefore, the model was age (years) = µ + herd + animal + error, where µ and herd were fixed effects, and animal was a random effect assumed to be normally distributed with mean = 0 and variance = 1. Herd was included in the model in an attempt to separate biological ageing (stress effect on methylation etc) from chronological ageing (the epigenetic clock component is not affected by stress). Excluding herd from the model decreased accuracy of age prediction substantially. The MRM was built in GCTA (Yang et al., 2011) and the model was fitted with variance components estimated in ASREML (Gilmour et al., 2015). Errors were assumed normally distributed.

To assess the accuracy of predicting age from the methylation profile, we used a cross validation strategy. Sets of 5 randomly chosen individuals had their phenotypes (ages) removed from the analysis, but they were still included in the MRM. This resulted in age effects being predicted for these animals. These age effects were then correlated with the actual age for these animals. The cross-validation procedure was repeated 10 times until all animals had been dropped from the analysis but included in the validation. The resulting correlation of predicted age and actual age was taken as the accuracy of prediction of age.

We also used a BayesR prediction method (Erbe et al., 2012) with Random Forest to impute missing genotypes (using the MissForest package in R (https://cran.r-project.org/web/packages/missForest/missForest.pdf), however this resulted in slightly worse accuracy of prediction compared with the MRM method (results not shown).

## Results

We performed 66 runs of MinION sequencing using untreated genomic DNA libraries. The resulting long sequence reads had an average mapping efficiency of 92.2% (SD = 2.8). The N50 size ranged from 1.09 Kb to 9.12 Kb across samples. To the best of our knowledge, this study also recorded the highest sequencing output generated from a single MinION flow-cell (41.13 GB, equivalent to 14.8-fold coverage; Table 1).

The number of methylation sites called for each of the 66 samples was variable, ranging from 2.8 to 17 M sites. At 3-fold coverage, up to 10 million methylation sites were detected; the highest number of methylation sites (17 M sites) were observed at 10-fold coverage. The number of observed methylation sites increased sharply with sequence coverage, and then plateaued at approximately 6-fold coverage (Figure 1C; Table 1).

**FIGURE 2:**
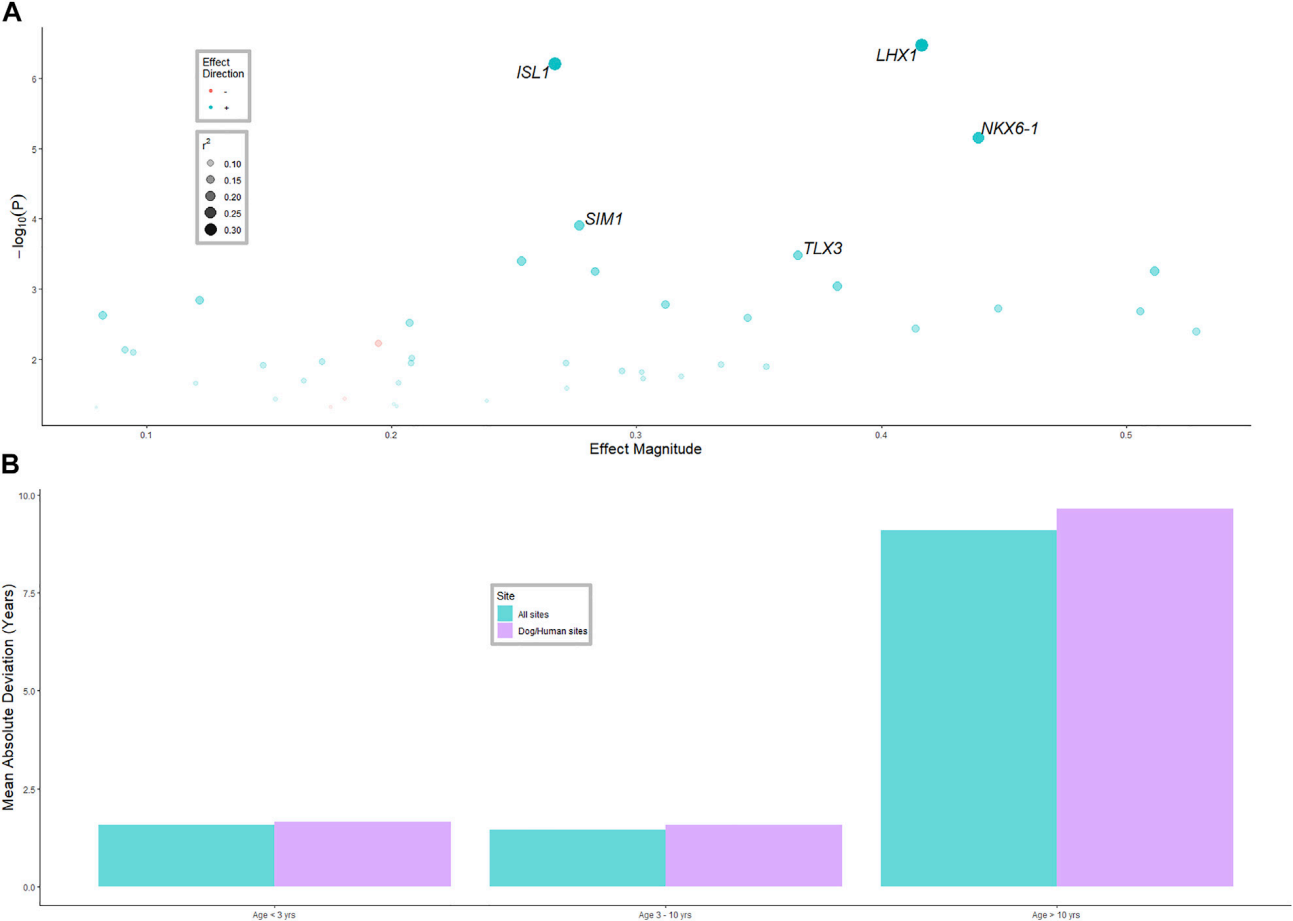
**(A)** Linear mixed model results for the model *age ∼ gene methylation + herd* (model was run separately for each gene, herd was random effect). The 43 significant (*p-*value < 0.05) genes that were associated with age in cattle, that were also associated with age in humans and dogs, are shown. The five most significant genes are labelled. See Supplementary Table S2 materials for all genes. **(B)** Average difference in age predicted from the methylation clock and actual age for three age classes of animals, and two methods of selecting methylated sites for inclusion in the clock.

Genes that were consistently associated with age in humans and dogs were tested for an association with age in cattle (Figure 2A). Of the 321 genes with sufficient data, 43 were associated with age in cattle, significantly more than expected by chance (*p-*value < 10^−6^). The *r* ^2^ values for these genes were mostly under 0.2, with NK6 Homeobox 1 (*NKX6-1)*, ISL LIM homeobox 1 (*ISL1)* and LIM homeobox 1 (*LHX1)* having *r* ^2^ values of (0.27, 0.35 and 0.36), respectively. Only three genes (*MAP4K3*, *MCF2L* and *SMAD7*) had a significant negative association with age, that is, they were de-methylated as the animals aged, while all other significantly associated genes were more methylated as the animals aged.

BLUP was used to predict age of animals from the whole genome methylation data, and from methylation data only near genes associated with age in humans and dogs. The correlation of predicted age and actual age in the validation datasets was 0.71 (moderate to high) for all methylation sites and 0.60 for the dog-human age associated sites; Figure 2B. The MAD was 1.4 years for animals aged less than 3 years and 1.5 years for animals aged 3–10 years. However, for animals from 10 years and older, the MAD was 9 years. The standard error of prediction accuracy was 0.08.

## Discussion

This study successfully sequenced and detected methylation sites from unmodified DNA extracted from hairs samples of 66 cattle using nanopore sequencing. We used the methylation data to identify genes associated with aging in cattle, and to predict age of animals from an industry relevant sample type.

This study achieved a prediction accuracy for age of 0.71. Considering the diverse genetic background of the animals sampled this accuracy is encouraging, that a usable epigenetic clock can be developed for extensively grazed cattle with diverse genetic backgrounds. Animals under 10 years old could be predicted within 1.5 years of age. With the addition of more animals into the model in the future the accuracy of the model may increase, especially in young animals where linear relationships between methylation and age have been reported in other species (Wang et al., 2020). Efforts to increase the number of methylation profiles in the reference dataset is ongoing, with the aim of increasing the accuracy of the clock for age prediction in cattle.

For animals above 10 years old the prediction accuracy for age was low. This is most likely because there were very few (*n* = 6) animals in the reference population that were older than 10 years. The result could also reflect a slower rate of methylation changes at more advanced ages (e.g., a non-linear rate of methylation; see Wang et al., 2020). Animals older than 10 years represent a very small class in the cattle industry and are the least likely to be candidates for age testing.

Our cattle epigenetic clocks captured the change in DNA methylation patterns as cattle age, similar to what has been previously observed in humans and other species. The number of samples used to derive our clock (*n* = 66) was higher than used in dogs and wolves (46 and 62 samples respectively, Thompson et al., 2017). Additionally, our study included methylation profiles overtime within individuals (*n* = 18), providing extremely valuable information (methylation changes within an individual) that could not be obtained by cross-sectional data from other epigenetic clocks. Our approach targeting sites overlapping with human and dog studies resulted in promising results, suggesting that epigenetic mechanisms are evolutionarily conserved, consistent with the findings of Lu et al. (2021).

The Nanopore sequencing used here may have some advantages over other methods used to detect methylation. At low coverage (3-fold), the number of methylation sites called by the ONT sequence data were 2.5 times higher than those called by reduced representation bisulfite sequencing (which captures around 4 million CpG dinucleotides from human samples; Gu et al., 2011). We observed a limited impact of coverage on total called sites, once coverage exceeded 6-fold. A study by Yuen et al. (2021) comparing methylation called by nanopore and whole genome bisulfite sequencing (WGBS) also noted that increasing coverage did not improve methylation prediction by both platforms. Other studies indicated that an average genomic coverage of 10 can accurately detect levels of methylation and haplotype using long-read nanopore sequencing (Gigante et al., 2019; Akbari et al., 2021). In contrast to ONT, WGBS, a standard platform for genome-wide identification of methylation, requires at least 30-fold coverage to cover all CpGs in the human genome (Stirzaker et al., 2014). This evidence, together with our results, suggest that ONT has the potential to offer low-cost genome-wide identification of methylation, SNPs, and structural variants with the ability to perform experiments in the field. Nanopore sequencing is also portable, which as discussed below opens up opportunities not possible with other methods.

As expected, only 13% (43) of the genes identified in Wang et al. (2020) were significantly associated with age in this study, confirming the different DNA methylation signatures across species and tissue types. Of note, tissue specific methylation differences have been reported (Zhang et al., 2013; Lowe et al., 2015; Zhou et al., 2020), which may also be a causal factor in the low validation rate. Five of these genes were significantly associated with age after a bonferroni correction for multiple testing, which is considered to be a highly conservitave correction method. In Wang et al., (2020) no correction for multiple testing was used, which may account for the low percentage of genes which were validated in this study. The shared genes could serve as promising candidates for the age prediction clock across species and for easy to obtain sample like hair.

The five most significant age associated genes were all transcription factors. *NKX6-1* (NK6 Homeobox 1), which plays a role in *β* cell function and proliferation (Avrahami et al., 2015) and has been found to have an association between methylation status and age (Iype et al., 2004); ISL1 which is a member of the LIM/homeodomain family of transcription factors and binds to the enhancer region of the insulin gene; ISL1 which has been reported to have an association between expression level and age (Tanizawa et al., 1994; Avrahami et al., 2015). *LHX1* which is a LIM domain homeobox transcription factor, associated with embryonic development (Costello et al., 2015); *SIM1* (SIM BHLH Transcription Factor 1); and *TLX3* (T Cell Leukemia Homeobox 3) which encodes a DNA-binding nuclear transcription factor. Transcription factors have been considered important in the regulation of genes which confer various biological functions associated with maturity and ageing (Roy, 1997). Increases in methylation of transcription factors as the individual ages could be the mechanism through which these genes are regulated over time.

Sequencing full genomes in-field for the purpose of age prediction is cost prohibitive, however a future approach may be to apply adaptive sequencing technology (Payne et al., 2020) or Cas9 target enrichment (McDonald et al., 2021) to enrich target regions in each sample that are efficient at predicting age. These methods would decrease the cost of sequencing each sample by 10- to 100-fold, making field application of methylation-based age prediction more economically viable for an industry setting, especially if it is combined with genomic prediction using the same data (Lamb et al., 2021). A single *in-situ* assay that can generate multiple types of desirable information for producers, such as animal age, genomic estimated breeding values, and parentage testing, is the ultimate goal. This may be achievable using ONT sequencing of samples that are routinely collected by industry, such as tail hair or ear punches.

This study has demonstrated that methylation patterns change as cattle age and that ONT sequencing is a useful tool for profiling methylation, as well as other genomic applications. The sample size of this dataset is small, and so the accuracy is expected to increase and improve with larger sample sizes. The cattle epigenetic clock from tail hair will allow extensively farmed beef production systems to develop the necessary tools to intensify genomic improvement and improve management outcomes.

## Data Availability

The datasets presented in this study can be found in online repositories. The names of the repository/repositories and accession number(s) can be found below: NCBI SRA BioProject, accession no: PRJNA770750.

## Abbreviations

MRM, methylation relationship matrix; MeBLUP, methylation best linear unbiased prediction; ONT, Oxford Nanopore Technology; MAD, The mean absolute deviation.
