# Supplementary figures and images for: An Epigenetic Aging Clock for Cattle Using Portable Sequencing Technology
Source: Front Genet. 2021 Nov 18;12:760450. doi: 10.3389/fgene.2021.760450 (PMC8637324; doi:10.3389/fgene.2021.760450)

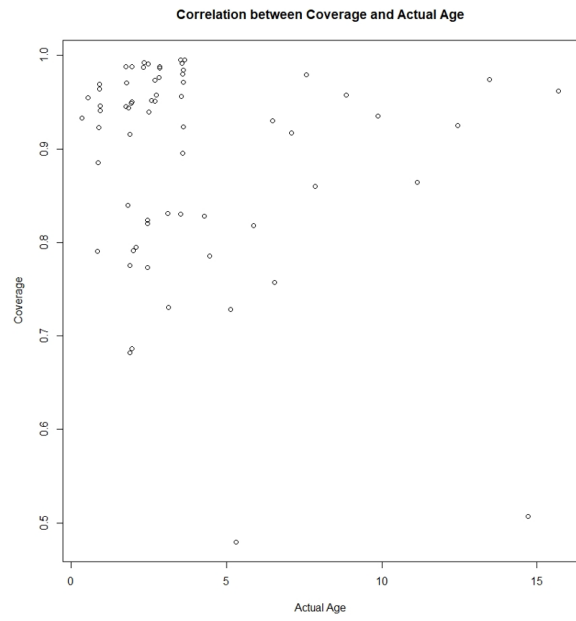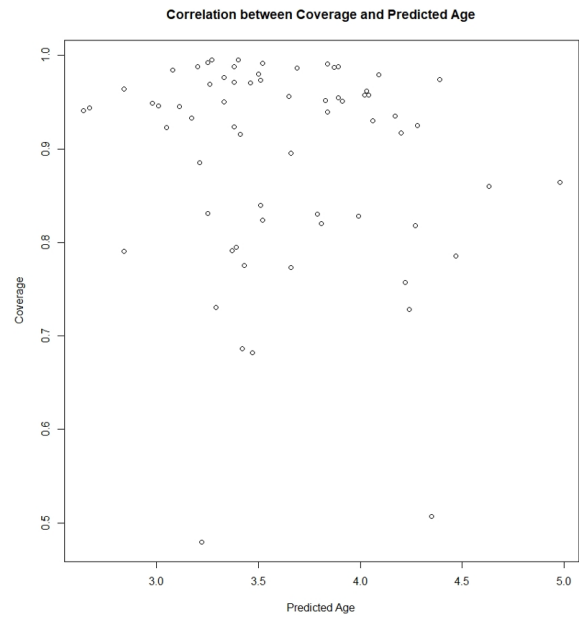

Supplement: Supplementary file 1 [file DataSheet1.PDF]
